# Monomerization of Cytosolic Mature Smac Attenuates Interaction with IAPs and Potentiation of Caspase Activation

**DOI:** 10.1371/journal.pone.0013094

**Published:** 2010-10-01

**Authors:** Stephen P. Burke, Jeffrey B. Smith

**Affiliations:** Department of Pharmacology and Toxicology, School of Medicine and Dentistry, University of Alabama at Birmingham, Birmingham, Alabama, United States of America; University of Texas MD Anderson Cancer Center, United States of America

## Abstract

The four residues at the amino-terminus of mature Smac/DIABLO are an IAP binding motif (IBM). Upon exit from mitochondria, mature Smac interacts with inhibitor of apoptosis proteins (IAPs), abrogating caspase inhibition. We used the ubiquitin fusion model to express mature Smac in the cytosol. Transiently expressed mature Smac56-239 (called Smac56) and Smac60-239 (called Smac60), which lacks the IBM, interacted with X-linked inhibitor of apoptosis protein (XIAP). However, stable expression produced wild type Smac56 that failed to homodimerize, interact with XIAP, and potentiate caspase activation. Cytosolic Smac60 retained these functions. Cytosolic Smac56 apparently becomes posttranslationally modified at the dimer interface region, which obliterated the epitope for a monoclonal antibody. Cytosolic Smacδ, which has the IBM but lacks amino acids 62–105, homodimerized and weakly interacted with XIAP, but failed to potentiate apoptosis. These findings suggest that the IBM of Smac is a recognition point for a posttranslational modification(s) that blocks homodimerization and IAP interaction, and that amino acids 62–105 are required for the proapoptotic function of Smac.

## Introduction

Human Smac/DIABLO is a cytoplasmically translated protein composed of 239 amino acids, the first 55 of which are required for mitochondrial import [1], [2]. The Smac gene consists of seven exons that can produce four isoforms: wild type Smac, Smacβ, Smacγ, and Smacδ [3], [4]. Wild type Smac lacks exon 2. Smacβ lacks exons 1 and 3, and translation of β initiates at an alternative start codon within exon 2, rendering it incompetent for mitochondrial translocation. Smacγ lacks exons 2 and 3, while Smacδ lacks exons 2 and 4. Upon translocation to the mitochondrial intermembrane space, an inner membrane peptidase complex removes the first 55 amino acids to produce mature Smac56 (Smac56-239) [5]. The first four amino acids of mature Smac (A^56^VPI^59^) are an IBM, which complexes with the BIR3 (baculovirus IAP repeat) domain of X-linked IAP (XIAP) [6], [7]. Homologous IBM sequences occur in *Drosophila* mitochondrial proteins Grim, Reaper, and Hid [7], the mitochondrial serine protease Omi/HtrA2 [8], and the p12 subunit of caspase-9 [9]. Structural studies of Smac complexed with the third BIR domain of XIAP suggested that the IBM may be essential for the interaction with IAPs [6], [10].

The Smac monomer is a double hairpin bundle of three α-helices [6]. Purified recombinant mature Smac forms an extraordinarily stable homodimer (half-life ∼20,000 years) [11]. The predominantly hydrophobic dimer interface forms an antiparallel four-helix bundle which has an arch shape [6]. The IBM of each Smac protomer can simultaneously interact with the second and third BIR domains of a single XIAP molecule [12]. Specific amino acid substitutions within the hydrophobic interface, such as F88D (also called F33D by subtraction of the first 55 residues), prevent Smac homodimerization [6]. The aforementioned Grim and Reaper have a GH3-like amphipathic helix, which is crucial to a proapoptotic function that is independent of IAP antagonism [13], [14]. Smacβ (also called Smac-S) and a truncated Smac76-239 mutant, both of which lack the IBM and localize to the cytosol, potentiated apoptosis evoked by chemotherapeutic agents [3], [15]. Importantly Smacβ, but not the truncated Smac76-239 mutant, complexed with XIAP, cIAP1, and cIAP2. While the IBM of Smac is not essential for the interaction with the IAPs, the segment close to the amino-terminus of mature Smac is necessary for IAP interaction [3].

There are eight human IAP family members, each of which has at least one BIR domain. The BIR domain, which is the defining feature of IAPs, is responsible for binding caspases. BIR2 and BIR3 of XIAP directly bind and inhibit processed capase-3 and processed caspase-9, respectively [reviewed in [16], [17]]. While XIAP binds and inhibits caspases, other IAPs seem not to directly inhibit the catalytic activity of caspases [17]. However, cIAP1 can potently prevent caspase-9 activation of procaspase-3 via interaction with the IBM of the p12 subunit of processed caspase-9 [18]. In addition to three BIR domains, XIAP has a RING domain with ubiquitin (Ub) ligase activity. Livin (also called ML-IAP) has a single BIR of the BIR3 type and a RING domain [19]. Survivin, the smallest member of the IAP family, has a lone BIR domain which may not bind IBMs [19]. cIAPs 1 and 2, have a CARD domain, which mediates protein interactions, three BIRs, and a RING domain. Apollon/BRUCE, the largest of the IAPs, has a single N-terminal BIR domain and a UBC (Ub conjugation domain) at the C-terminus [20]. The two remaining IAPs, testis specific IAP (Ts-IAP) and neuronal apoptosis inhibitory protein (NAIP), were not included in the present study. Transcripts of all the IAPs, except Ts-IAP and NAIP, were detected by RT-PCR in the cell model used here, namely the 911 line of human embryonic retinoblasts (A. D. Steg and M. R. Johnson, unpublished data, University of Alabama at Birmingham).

IAPs with a RING domain dock an E2 Ub conjugating enzyme and a target protein that accepts Ub, which constitutes an E3 Ub ligase [21], [22], [23]. cIAPs 1 and 2, Livin, and XIAP catalyze transubiquitination of substrate proteins, such as cytosolic mature Smac and caspases [24], [25], [26]. The converse can also occur, for example, Smac can selectively reduce the levels of cIAP1 and 2 by promoting their autoubiquitination [27]. Besides transubiquitinating substrate proteins, XIAP also is subject to autoubiquitination [28]. Although Apollon lacks a RING domain, it uses an E2 Ub conjugation domain to ubiquitinate Smac and caspase-9 [29]. Grim and Reaper, which are functional homologs of Smac, can stimulate autoubiquitination of XIAP [30], [31]. Coexpression of ectopic Smacδ and XIAP decreased the cellular level of endogenous XIAP [4]. cIAPs play important roles in the regulation of tumor necrosis factor alpha (TNFα)-mediated NF-κB activation [32], [33], [34]. cIAPs are multifunction E3 Ub ligases which can polyubiquitinate target proteins via Lys-48 or Lys-63 of Ub. The latter serves a nondegradative function. For example, by addition of a Lys-63-linked polyubiquitin chain to RIP1, cIAPs influence TNFα -induced NF-κB activation [35], [36]. IAPs clearly regulate apoptosis by multiple mechanisms, which are subject to modulation by Smac.

In this study, we used the Ub fusion model to determine whether wild type mature Smac maintains a proapoptotic function after prolonged cytosolic localization, and evaluate the requirement of the IBM to induce apoptosis [37], [38], [39]. We show that stable cytosolic expression of Smac56 attenuates homodimerization and Smac's ability to bind IAPs, which are considered critical for the proapoptotic function of Smac. The present study has uncovered a novel mechanism of Smac downregulation, which is distinct from protein disassembly by the Ub proteasome system.

## Results

### Ub fusion produces mature Smac in the cytosol after stable or transient transfection

**Figure 1:**
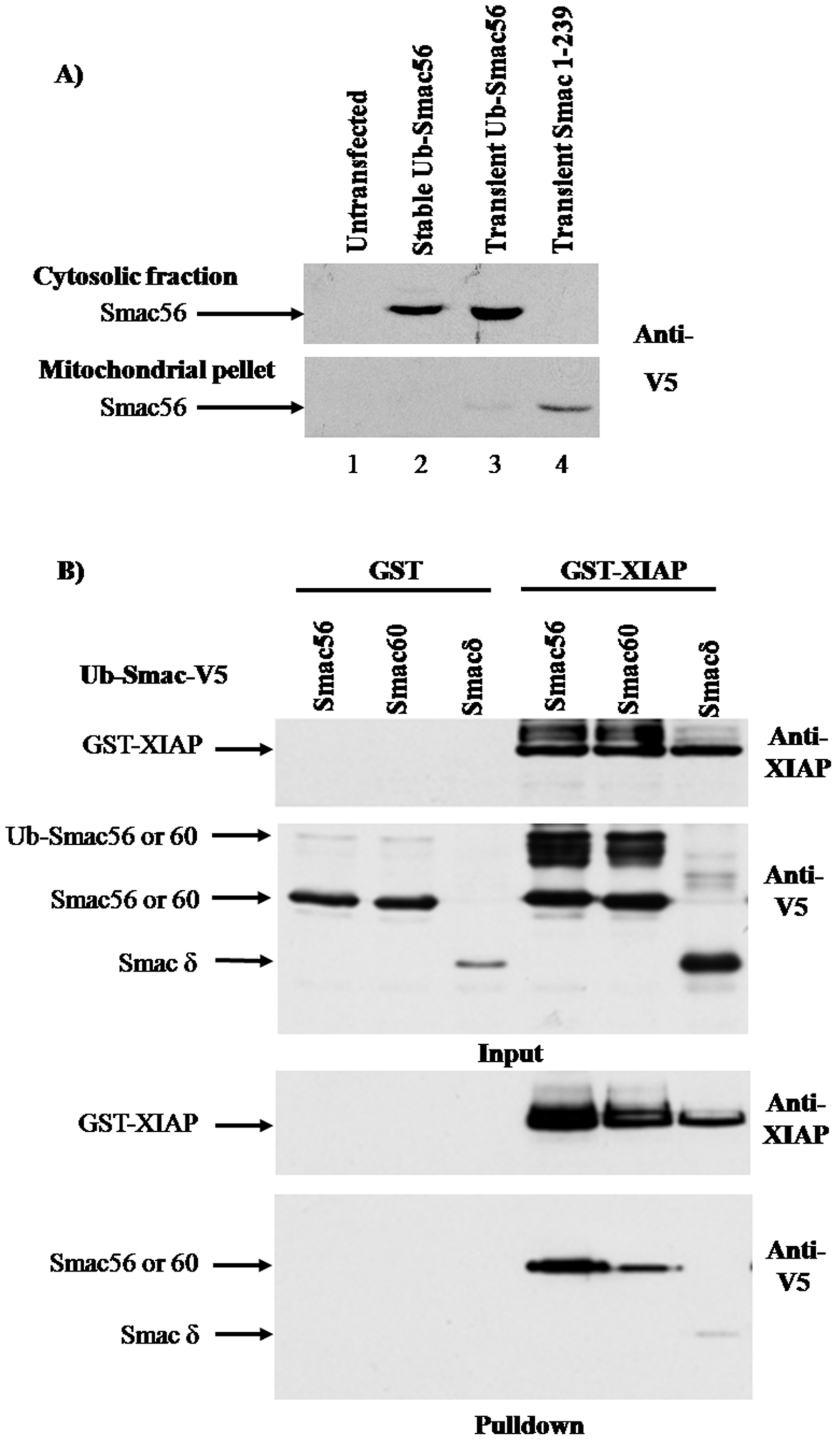
Cytosolic expression of mature Smac variants and the interaction of Smac56, Smac60, and Smacδ with XIAP following transient expression. (**A**) Cells were stably or transiently transfected with IRES2.eGFP expression plasmid (*Stable* versus *Transient*) containing Ub-Smac56 or full-length Smac 1-239. 911 cells were subjected to hypotonic lysis, and cytosol and mitochondrial fractions were prepared by differential centrifugation. Proteins were resolved by SDS-PAGE and subjected to western blot analysis with a V5 antibody to detect ectopic Smac. (**B**) Cells were transiently cotransfected with the indicated GST vector (pGFLEX) and the indicated HA-Ub-Smac (pcDNA3.1-V5His). Two days later cells were lysed, and proteins were purified using GSH-agarose. Lysate proteins (*Input*) and proteins eluted from the GSH-agarose (*Pulldown*) were resolved by SDS-PAGE and subjected to western blot analysis with antibody to the V5 epitope or XIAP.

Mammalian cells have the highly specific protease, ubiquitin C-terminal hydrolase (UCH), which cotranslationally cleaves the nascent polyubiquitin protein between Gly 76 of one Ub and the initial Met of the next Ub or removes Ub when it is fused to the amino-terminal residue of another protein [37], [38]. Smac^1-239^-V5 and Ub-Smac56-V5 fusion constructs were stably or transiently expressed in 911 cells. Western blot analysis of mitochondrial and cytosol fractions, confirmed cytosolic expression of Smac56-V5 following stable or transient expression (Fig. 1 A, lanes 2 and 3). In contrast, ectopic expression of full-length Smac^1-239^-V5, which has the mitochondrial localization sequence (the first 55 residues), produced mature Smac56-V5 that was predominantly in the mitochondrial pellet (Fig. 1A, lane 4). The minor amount of transient Smac56-V5 from the Ub fusion protein in the mitochondrial fraction was likely due to slight contamination with cytosol (Fig. 1A, lane 3).

### Interaction of transiently expressed Smac variants with XIAP

911 cells were transiently cotransfected with GST-XIAP and wild type or a variant of Ub-Smac-V5 in order to evaluate the ability of Smac to interact with XIAP. GST-XIAP pulled down wild type Smac56 and Smac60, which lacks the IBM. The pulldown of Smac60 was slightly less efficient than the pulldown of wild type Smac56-V5 (Fig. 1B, lanes 4 and 5). Smacδ-V5, which has the IBM but lacks amino acids 62 to 105, was scarcely detectable in the GST-XIAP pulldown by comparison to the pulldown of Smac56-V5 or Smac60-V5 (Fig. 1B, lane 6). These results indicate that the IBM of Smac is neither sufficient, nor necessary, for interaction with XIAP, as reported previously [3], [6].

There was no detectable Smac pulled down from cells that were transfected only with Ub-Smac56-V5 or Smac60-V5, which confirmed the requirement for GST-XIAP (Fig. 1B, lane 1 and 2).

### Homo- and hetero-dimerization of transiently expressed wild type Smac56 and Smacδ

**Figure 2:**
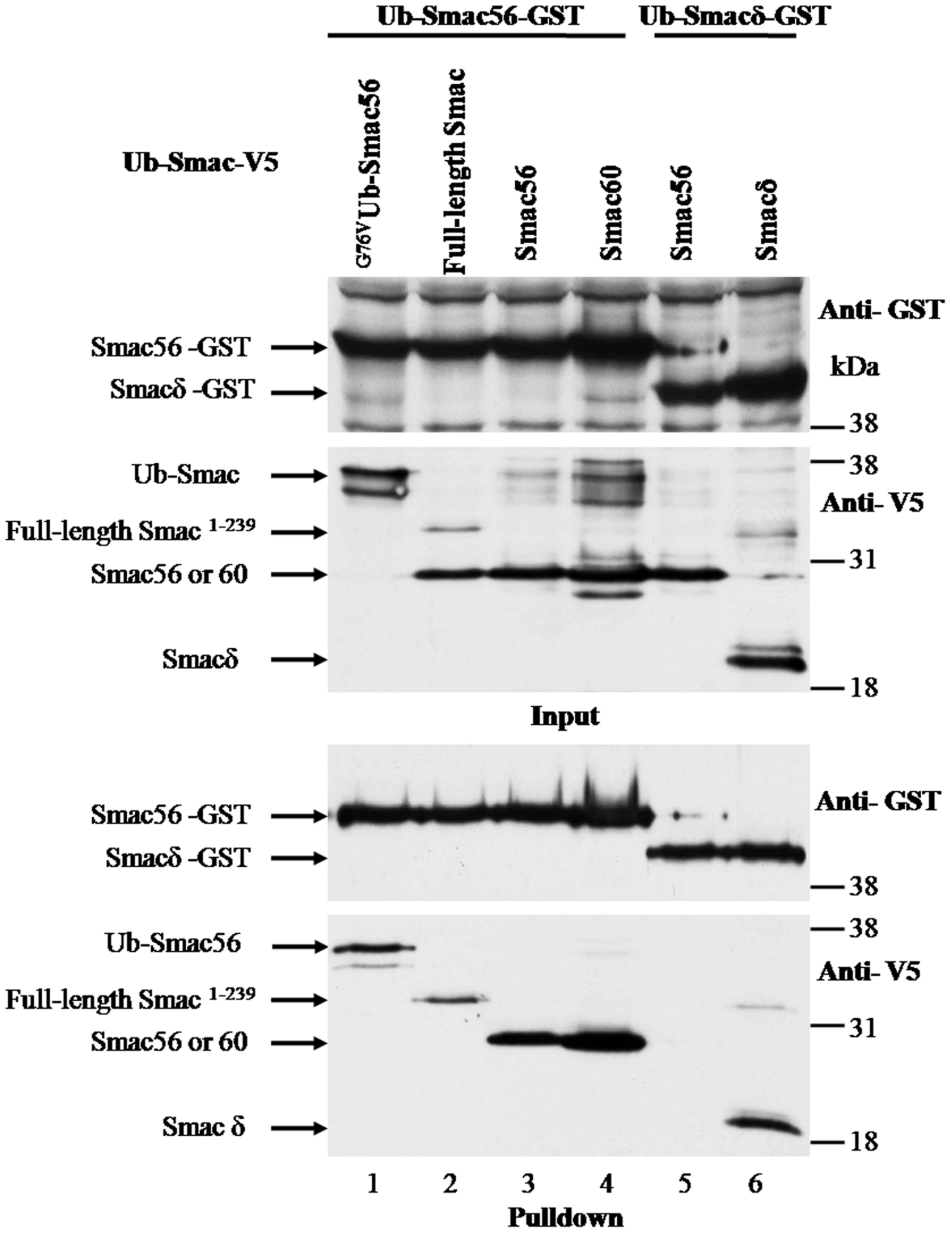
Transiently expressed Smac56 homodimerizes and heterodimerizes with Smac60, but not with Smacδ. Cells were transiently cotransfected with the Ub-Smac56 or Ub-Smacδ with a C-terminal GST tag (pGFLEX) and with full-length Smac^1-239^, Ub-Smac56, Ub-Smac60, Ub-Smacδ, or ^G76V^Ub-Smac56 with a C-terminal V5 tag (pcDNA3.1-HA-Ub-Smac-V5), as indicated. Cells were lysed, and proteins were purified using GSH-agarose. Lysate proteins (*Input*) and proteins eluted from the GSH-agarose (*Pulldown*) were resolved by SDS-PAGE and subjected to western blot analysis with antibody to GST-pi and to the V5 tag.

Pulldown experiments were done with lysates from cells that transiently coexpressed ectopic Ub-Smac56 or Ub-Smacδ with a C-terminal GST tag and wild type Ub-Smac56 or a Ub-Smac variant with a C-terminal V5 tag. Smac56-GST interacted strongly with Smac56-V5 and with Smac60-V5, as indicated by abundance of Smac-V5 in the pulldown (Fig. 2, lanes 3 and 4). Expression of full-length Smac^1-239^-V5 produced mature Smac56-V5 (Input, anti-V5 panel) as a result of processing within mitochondria and the band of unprocessed Smac^1-239^-V5, which presumably had not yet been imported by mitochondria (Fig. 2, lane 2). Interestingly, Smac56-GST pulled down unprocessed full-length Smac^1-239^-V5, but the mitochondrially processed Smac56-V5 was undetectable in the pulldown (Fig. 2, lane 2). This result suggests that the mitochondrial Smac56-V5 may be present as a homodimer that is too stable to exchange with cytosolic Smac-56-GST homodimer during the experiment. Consistent with the pulldown of full-length Smac^1-239^, Smac56-GST pulled down unprocessed ^G76V^Ub-Smac56-V5 fusion protein (Fig. 2, lane 1). Thus, neither the first 55 amino acids of Smac, nor Ub fused to the amino-terminus of Smac prevented Smac dimerization. Although Smacδ-GST pulled down Smacδ-V5, Smacδ-GST failed to pulldown detectable wild type Smac56-V5 (Fig. 2, lanes 5 and 6). Thus, while Smacδ homodimerized, it failed to heterodimerize with wild type Smac56. This result suggests that the amino acids 62 to 105 of wild type Smac prevent it from interacting with Smacδ.

### Cytosolic Smac60 potentiates PARP cleavage and caspase activation evoked by proteasome blockade

**Figure 3 pone-0013094-g003:**
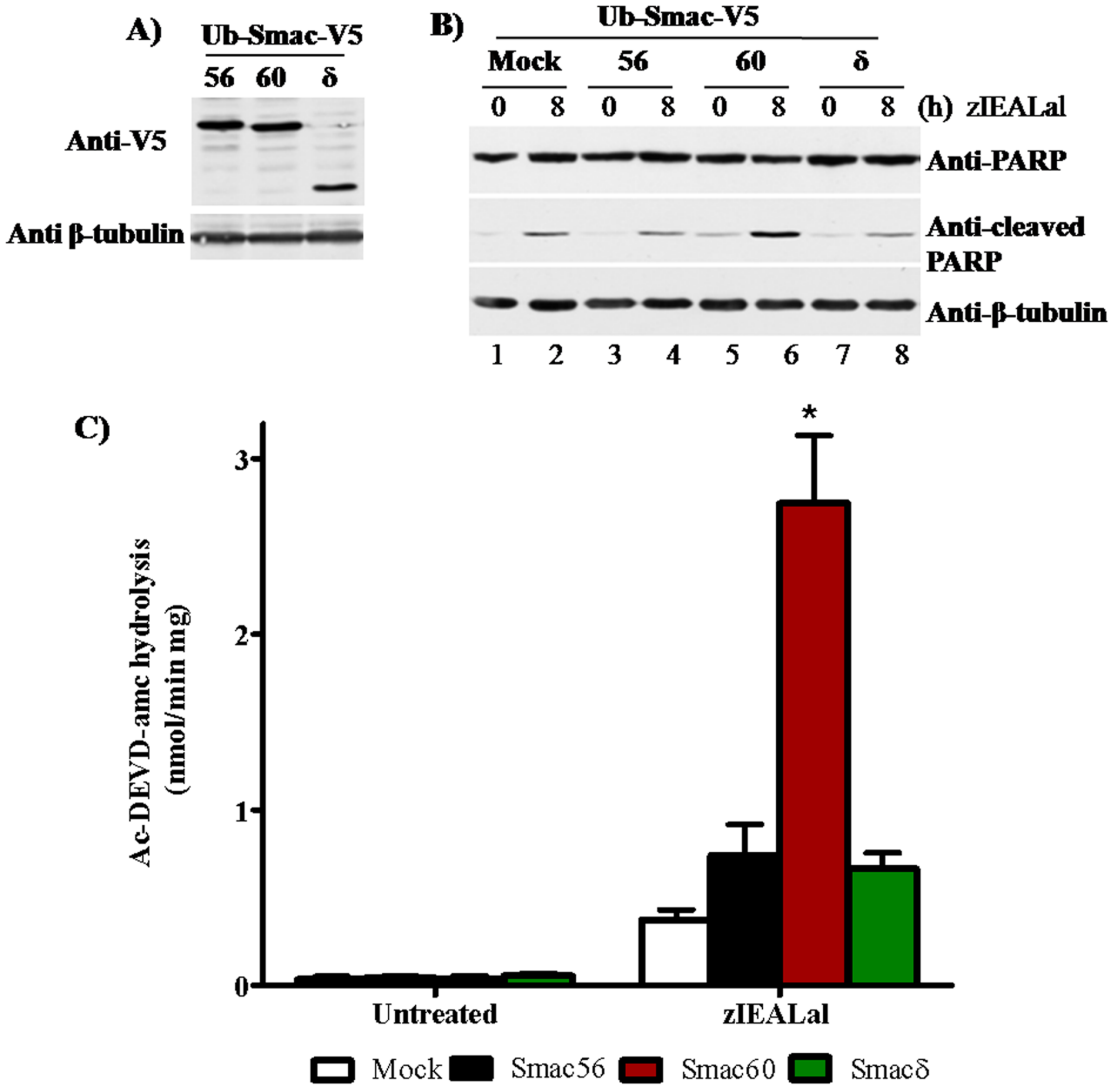
Persistent cytosolic expression of Smac60, but not Smac56, potentiates activation of caspase-3-like activity by zIEALal. (**A**) Cells, which stably expressed the indicated Smac variant in the IRES2.eGFP vector, were lysed and proteins (30 µg) were subjected to western blot analysis with V5 antibody. (**B**) Stable Smac cell lines (IRES2.eGFP) were plated, and the following day they were treated for 8 h with 20 µM zIEALal or left untreated. Cell lysates were subjected to western blot analysis for intact PARP (B, top panel) or PARP cleaved after aspartate 214 (B, middle panel). Anti β-tubulin western blot confirmed equivalent protein loading (B, bottom panel). (**C**) Cell lines were treated for 18 h with 20 µM zIEALal or untreated. Lysates (0.75 µg) were assayed for caspase-3-like activity by continuously recording fluorescence of amc produced by hydrolysis of Ac-DEVD-amc. Columns indicate mean amc produced ± S.E.M. Two-way Anova followed by the Bonferroni post-test gave a p value of <0.001 for zIEALal treated Smac60 compared to the empty IRES vector (n = 4) (Prism 5, Graphpad Software). *Mock* indicates cell lines stably transfected with empty vector.

**Figure 4 pone-0013094-g004:**
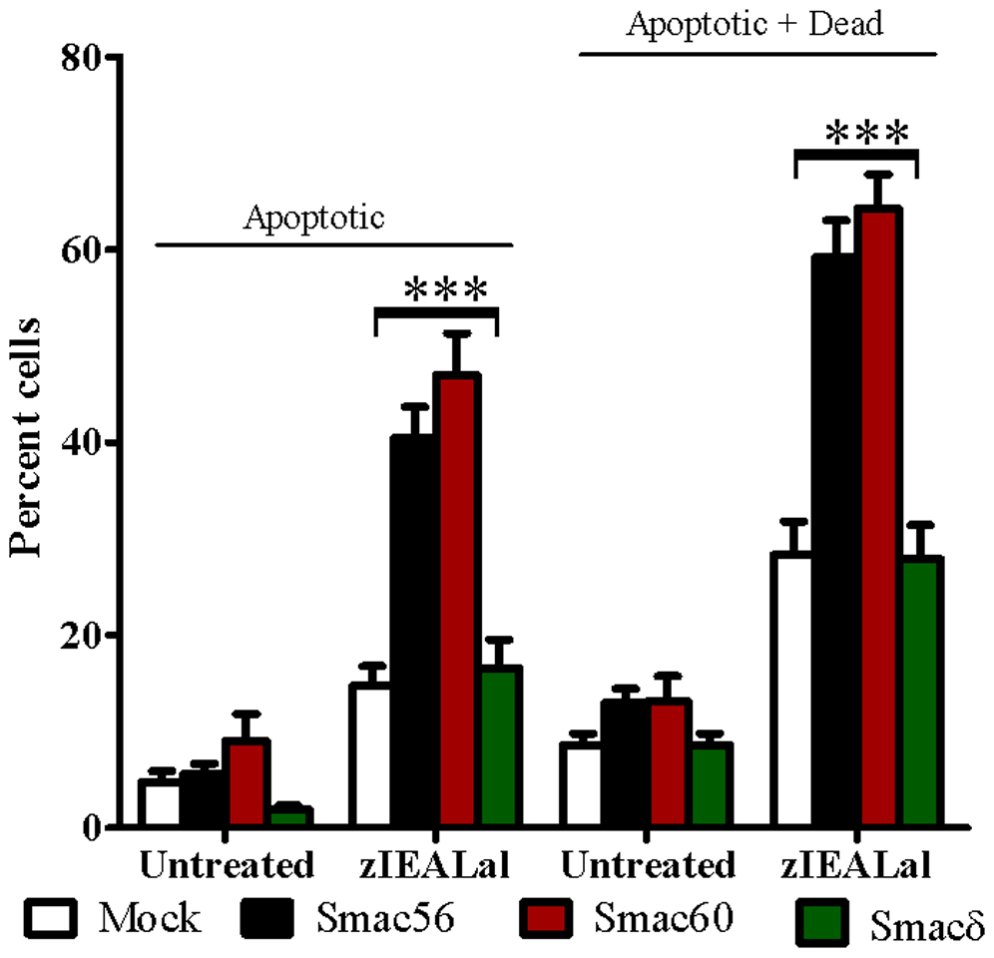
Persistent cytosolic expression of Smac56 or Smac60 potentiates apoptosis and cell death evoked by zIEALal. Cell lines that stably expressed the indicated Smac variant (3 independent clones transfected with empty vector (IRES2.eGFP) (*Mock*) or with the indicated Smac variant) were incubated with or without zIEALal for 24 h. Following the treatment, both floating and attached cells were harvested and stained with annexin V-PE and 7-AAD, and analyzed by FACS. Apoptotic cells positively stained for annexin V-PE only, and dying cells were positively stained for 7-AAD only or for 7-AAD and annexin V-PE. Values are mean ± S.E.M (n = 9). Two-way Anova followed by the Bonferroni post-test gave the indicated p values (***<0.001) (Prism, Graphpad Software).

Next we compared the degree of PARP processing evoked by proteasome blockade in cell lines that expressed cytosolic Ub-Smac-V5 variants (Smac56, Smac60, and Smacδ) or empty vector (IRES2.eGFP). Western analysis showed that the cell lines expressed similar levels of wild type and Smac variant proteins (Fig. 3A). The cells were treated with a potent peptidyl proteasome inhibitor, zIEALal, for 8 h or were untreated. Faint levels of cleaved PARP were detectable in untreated cells. Treatment with zIEALal increased cleaved PARP in lysates of cells that expressed GFP only (“Mock”), Smac56-V5, or Smacδ-V5 (Fig. 3B, lanes 2, 4, and 8). Surprisingly, Smac60-V5 markedly potentiated the accumulation of cleaved PARP (Fig. 4B, lanes 5 and 6). Thus, Smac60, but not wild type or Smacδ, potentiated the induction of apoptosis evoked by proteasome blockade.

Treatment with zIEALal for 18 h increased hydrolysis of caspase-3 substrate Ac-DEVD-amc to varying extents in lysates of cells that expressed a cytosolic Smac-V5 variant (Smac56, Smac60, and Smacδ) or empty vector (“Mock”) (Fig. 3C). Smac60 and produced a 7.3 fold potentiation of caspase-3-like activity evoked by zIEALal. Smac56 and Smacδ only modestly increased caspase-3 like activity (1.7- and 1.5-fold, respectively) relative to the empty vector (Fig. 3C).

### Cytosolic Smac56 and Smac60 potentiate induction of apoptosis and cell death

Apoptosis and cell death were determined by flow cytometry analysis of annexin V-PE binding and 7-AAD uptake by cell lines expressing cytosolic Smac56, 60, and δ, after a 24 h zIEALal treatment (Fig. 4). Stable expression of cytosolic Smac56 and Smac60, but not Smacδ, potentiated the induction of apoptosis and cell death evoked by zIEALal. Smac56 and Smac60 potentiated the induction of apoptosis by 2.8- and 3.2-fold, respectively, relative to the cells that expressed only GFP (“Mock”) (Fig. 4, “Apoptotic”). The much greater potentiation of caspase-3 activation (Fig. 3C), but similar induction of apoptosis by Smac60 compared to wild type Smac56 suggests that caspase activation may not mediate the proapoptotic action of cytosolic Smac56.

### Stably expressed cytosolic Smac56 fails to dimerize or interact with XIAP

**Figure 5:**
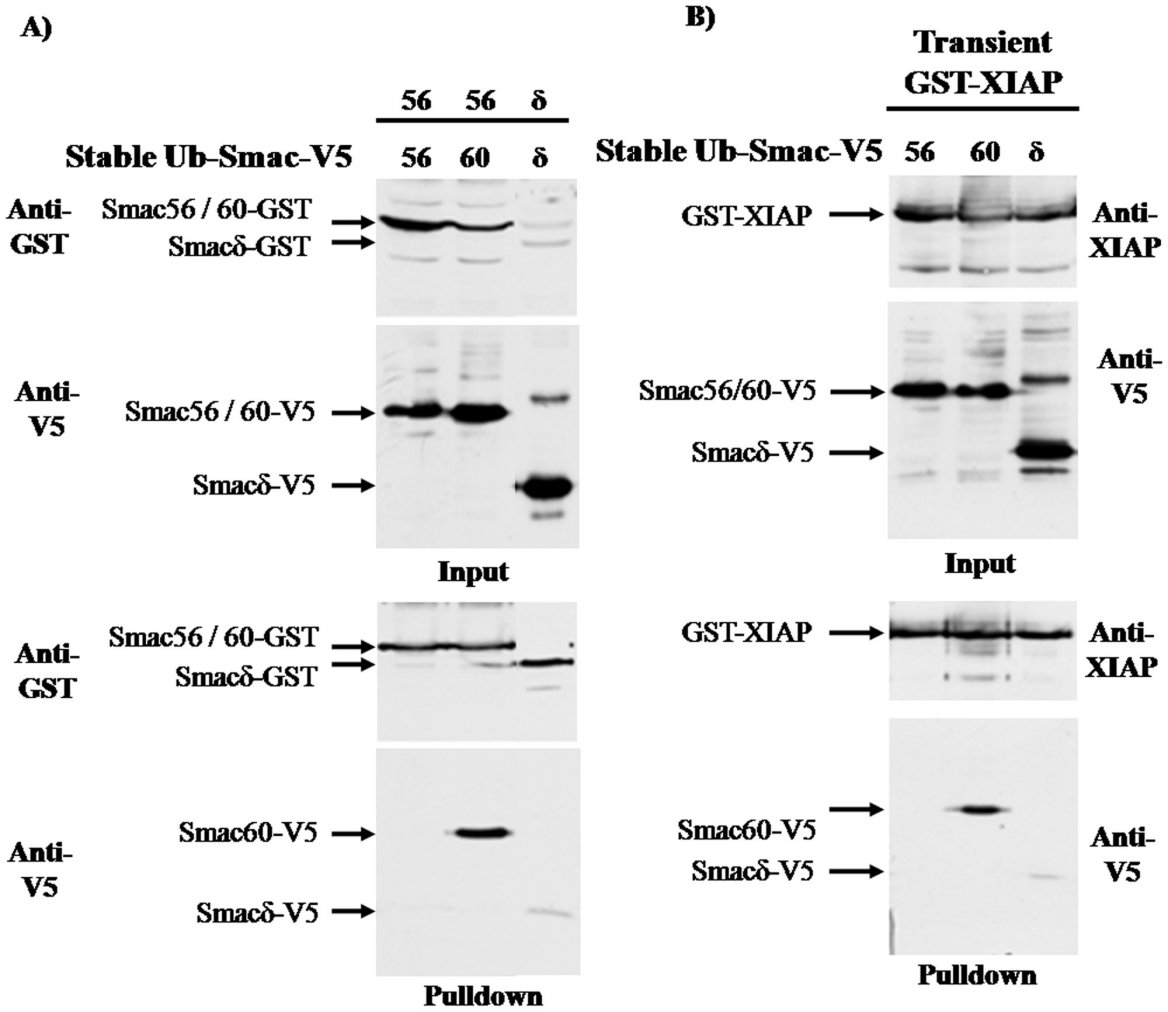
Persistent cytosolic expression of Smac56, but not Smac60, ablates dimerization and interaction with XIAP. (**A**) Cells that stably expressed the indicated Ub-Smac-V5 plasmid (IRES2.eGFP) were transiently transfected with the Ub-Smac-GST plasmids (pGFLEX), lysed, and proteins were purified using GSH-agarose. Lysate proteins (*Input*) and proteins eluted from the GSH-agarose (*Pulldown*) were resolved by SDS-PAGE and subjected to western blot analysis with antibody to GST-pi and the V5 epitope. (**B**) Cells that stably expressed the indicated Ub-Smac-V5 plasmid (IRES2.eGFP) were transiently transfected with GST-XIAP plasmid (pGFLEX), lysed, and *Input* and *Pulldown* proteins were resolved by SDS-PAGE and subjected to western blot analysis with antibody to XIAP and the V5 epitope as described for (A).

Cell lines that stably expressed cytosolic Smac56-V5, Smac60-V5, and Smacδ-V5 were transiently transfected with Ub-Smac56 or Ub-Smacδ with a C-terminal GST tag. The cells were lysed and proteins were pulled down with GSH agarose to assess the capacity of Smac to dimerize. Smac56-GST pulled down stably expressed Smac60-V5, but no Smac56-V5 was detectable in the pulldown (Fig. 5A, lower panel). Smacδ-GST pulled down Smacδ-V5, but to a much smaller extent than the pulldown of Smac60-V5 by Smac56-GST (Fig. 5A). Furthermore the cell lines that stably expressed Smac56-V5, Smac60-V5, and Smacδ-V5 were transiently transfected with GST-XIAP to determine whether the cytosolic Smac variants interacted with XIAP. Consistent with the failure of stably expressed Smac56 to dimerize, GST-XIAP failed to pulldown stably expressed Smac56 (Fig. 5B). GST-XIAP readily pulled down stably expressed Smac60 and, as previously reported [4], weakly pulled down Smacδ (Fig. 5B). These results indicate that persistent cytosolic expression of Smac56, severely impairs the ability of Smac56, but not Smac60, to dimerize and to interact with XIAP.

### Smac56 fails to oligomerize after stable cytosolic expression

**Figure 6 pone-0013094-g006:**
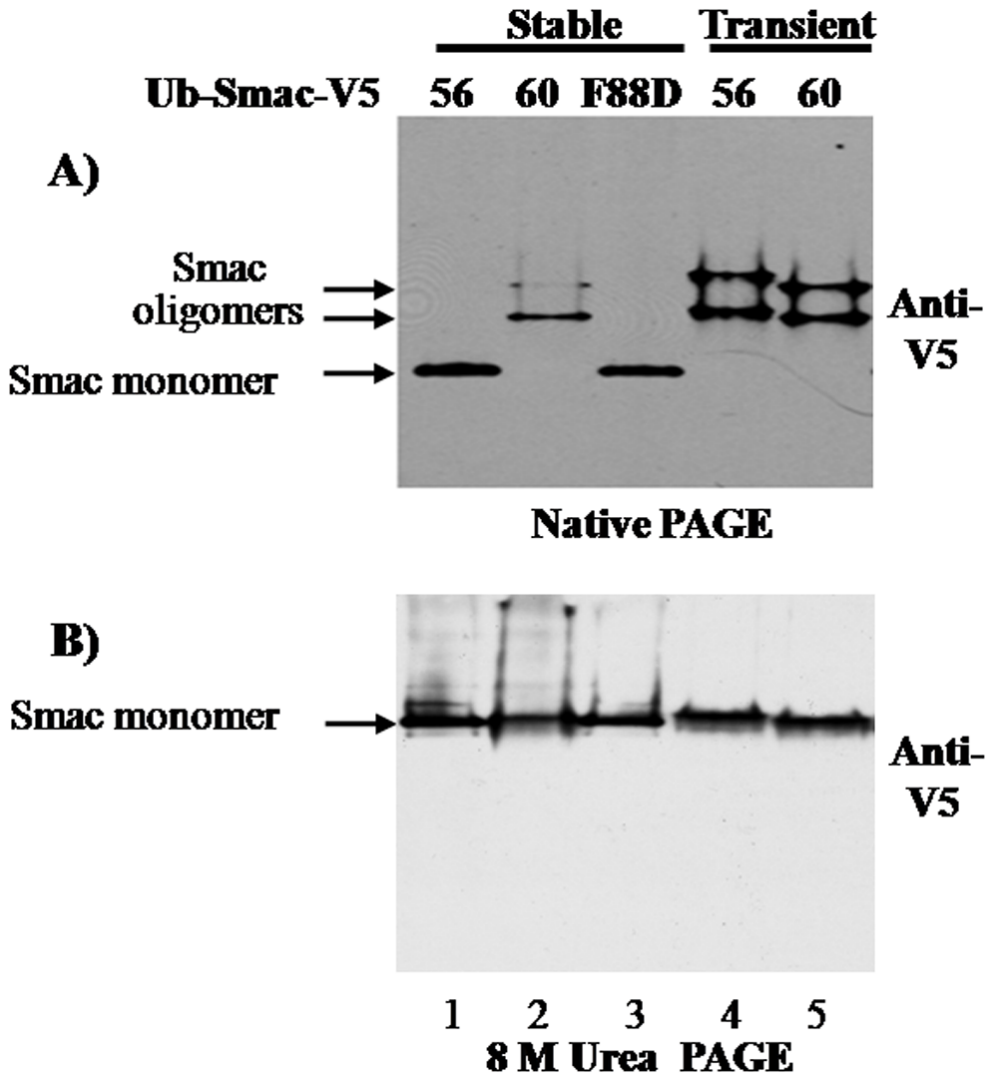
Native and urea PAGE of Smac56, Smac60, and the dimer interface F88D mutant of Smac56. Cell lines that stably expressed the indicated Ub-Smac-V5 (IRES2.eGFP) and cells that were transiently transfected with Ub-Smac56-V5 or Ub-Smac60-V5 (IRES2.eGFP) were lysed, and proteins were resolved by native PAGE or by PAGE in the presence of 8 M urea as indicated. Western analysis was done with antibody to the V5 epitope.

Recombinant Smac forms an elongated dimer, which elutes as an approximately 100 kDa species by gel filtration chromatography, while the F88D missense mutant of mature Smac is a 21 kDa monomer [1], [6]. Proteins from cells that transiently or stably expressed cytosolic Smac56-V5, Smac60-V5, or ^F88D^Smac56-V5 were fractionated by native or 8 M urea PAGE. Ectopic Smac56-V5 and Smac60-V5 have a predicted charge of minus 11 at pH 7, which explains the mobility during native electrophoresis. Under native conditions, stably expressed Smac56-V5 and ^F88D^Smac56-V5 migrated as a single band with essentially the same mobility (Fig. 6, lanes 1 and 3). Transiently expressed Smac56-V5, like stably and transiently expressed Smac60-V5, migrated as two bands with reduced electrophoretic mobility compared to the monomeric ^F88D^Smac56-V5 mutant (Fig. 6A, lanes 2, 4, and 5). The two Smac bands with reduced mobility are probably oligomeric species, such as a dimer and tetramer, because they migrated as a single species in the presence of 8 M urea and comigrated with monomeric ^F88D^Smac56-V5 (Fig. 6, bottom). Because Smac migrated as a single band in the presence of 8 M urea, the two Smac bands observed by native PAGE chiefly differ in mass, presumably in quaternary structure, rather than in charge.

**Figure 7 pone-0013094-g007:**
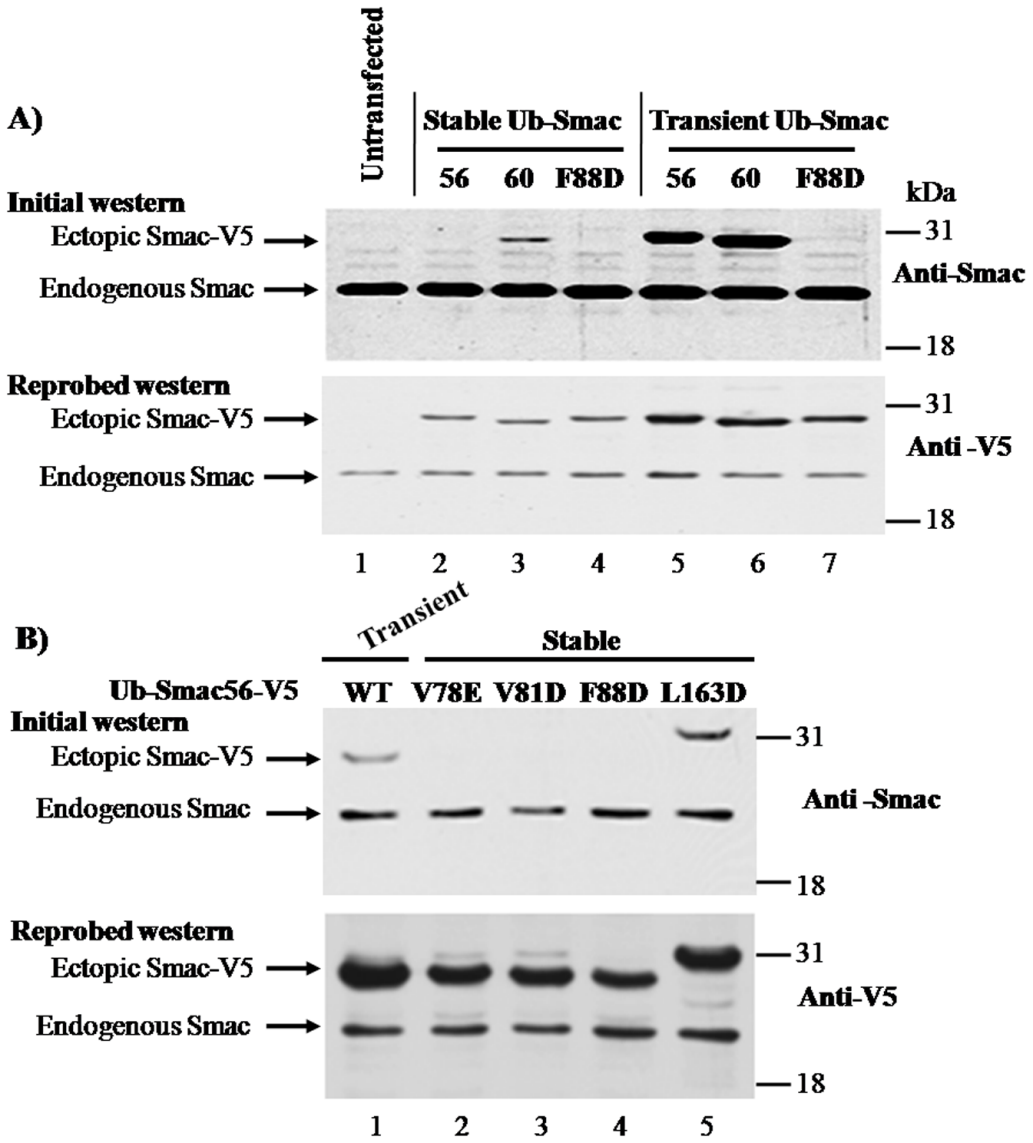
Persistent cytosolic expression of Smac56, but not Smac60, ablates immunostaining by a monoclonal antibody similarly to certain missense mutations of the dimer interface. (**A**) Cell lines that stably expressed the indicated Ub-Smac-V5 plasmids (IRES2.eGFP) and cells that were transiently transfected with Ub-Smac56-V5 or Ub-Smac60-V5 (IRES2.eGFP) were lysed and proteins were resolved by SDS-PAGE. *Mock* indicates a cell line transfected with empty IRES2.eGFP vector. Western blot analysis was done firstly with the monoclonal Smac antibody and secondly with antibody to the V5 epitope. (**B**) Cell lines that stably expressed the indicated missense mutation of Ub-Smac56-V5 (IRES2.eGFP) and cells that were transiently transfected with Ub-Smac56-V5 (IRES2.eGFP) were lysed, and proteins were resolved by SDS-PAGE. Western blot analysis was done as indicated for (A).

On the native gel, the two bands of stably or transiently expressed Smac60-V5 had a slightly faster mobility than those of transiently expressed Smac56-V5 (Fig. 6, top). Similarly, Smac60-V5, which is ∼400 Da smaller than Smac56-V5, migrated slightly faster than wild type Smac56-V5 by SDS-PAGE (Fig. 7A, anti-V5, lanes 2, 3, 5, and 6). These findings indicate that persistent expression of wild type Smac56, but not Smac60, in the cytosol of 911 cells essentially abolished its ability to oligomerize. Hence, persistent cytosolic expression of wild type Smac56, but not Smac60, predisposes it to a posttranslational modification(s) that blocks dimer formation without affecting its mobility by SDS-PAGE (Fig. 7A, anti-V5, lanes 2 and 5) or 8 M urea PAGE (Fig. 6B, lanes 1 and 4).

### Monoclonal Smac antibody fails to detect ectopic wild type Smac56 after stable cytosolic expression

The following results show that presistent cytosolic expression of wild type Smac56 essentially abolished immunostaining by a monoclonal Smac antibody. The antibody failed to immunostain Smacδ, which lacks amino acids 62–105 (Burke and Smith, unpublished data). The epitope of the Smac antibody was further localized to a dimer interface region of Smac using Smac mutants (Fig. 7). Proteins from cells that stably or transiently expressed cytosolic, V5 tagged Smac56, Smac60, or ^F88D^Smac56 and untransfected cell lysates were resolved by SDS-PAGE, and analyzed by successive western blots with a monoclonal antibody to Smac or to the V5 tag. The Smac monoclonal antibody immunostained endogenous mitochondrial Smac56 and transiently expressed Smac56-V5 and Smac60-V5, which migrated somewhat slower than endogenous wild type Smac56 due to the presence of the V5 epitope tag (Fig. 7A). The Smac monoclonal antibody failed to immunostain stably expressed Smac56-V5 or the following dimeric interface mutants of Smac56: V78E, V81D, or F88D Smac56-V5 after stable or transient expression (Fig. 7A and 7B). Similarly the Smac monoclonal failed to recognize the V78E, V81D, and F88D Smac56 mutants following transient expression in 911 cells (Burke and Smith, unpublished data). The Smac antibody immunostained transiently expressed Smac56-V5, stably or transiently expressed Smac60-V5, and the stably expressed L163D Smac56 mutant (Fig. 7A and 7B). The L163D mutant fails to dimerize [6]. Note that there was less ectopic wild type Smac56 and Smac60 after stable compared to transient expression (Fig. 7A, lanes 3 and 6, anti-Smac and anti-V5 panels). Note also that the level of endogenous Smac was similar to that of ectopic Smac after transient expression (Fig. 7A, anti-Smac). Thus the cellular level of ectopic Smac after stable expression was substantially lower than that of endogenous Smac (Fig. 7A).

Wild type and mutant Smac proteins were similarly immunoreactive with the antibody to the V5 tag (Fig. 7A and 7B). These results suggest that a posttranslational modification(s) at or near the dimer interface, which includes residues V78 to F88, is responsible for the loss of immunoreactivity. Smac dimerization per se does not affect the integrity of the epitope because the antibody immunostained the L163D mutant (Fig. 7B, anti-Smac). Furthermore, native PAGE of L163D Smac56 showed that it migrated as a single band with essentially the same mobility as the F88D mutant (Burke and Smith, unpublished data).

### Stable cytosolic expression of Smac56 decreases its interaction with Apollon and cIAP1 and cIAP2

**Figure 8:**
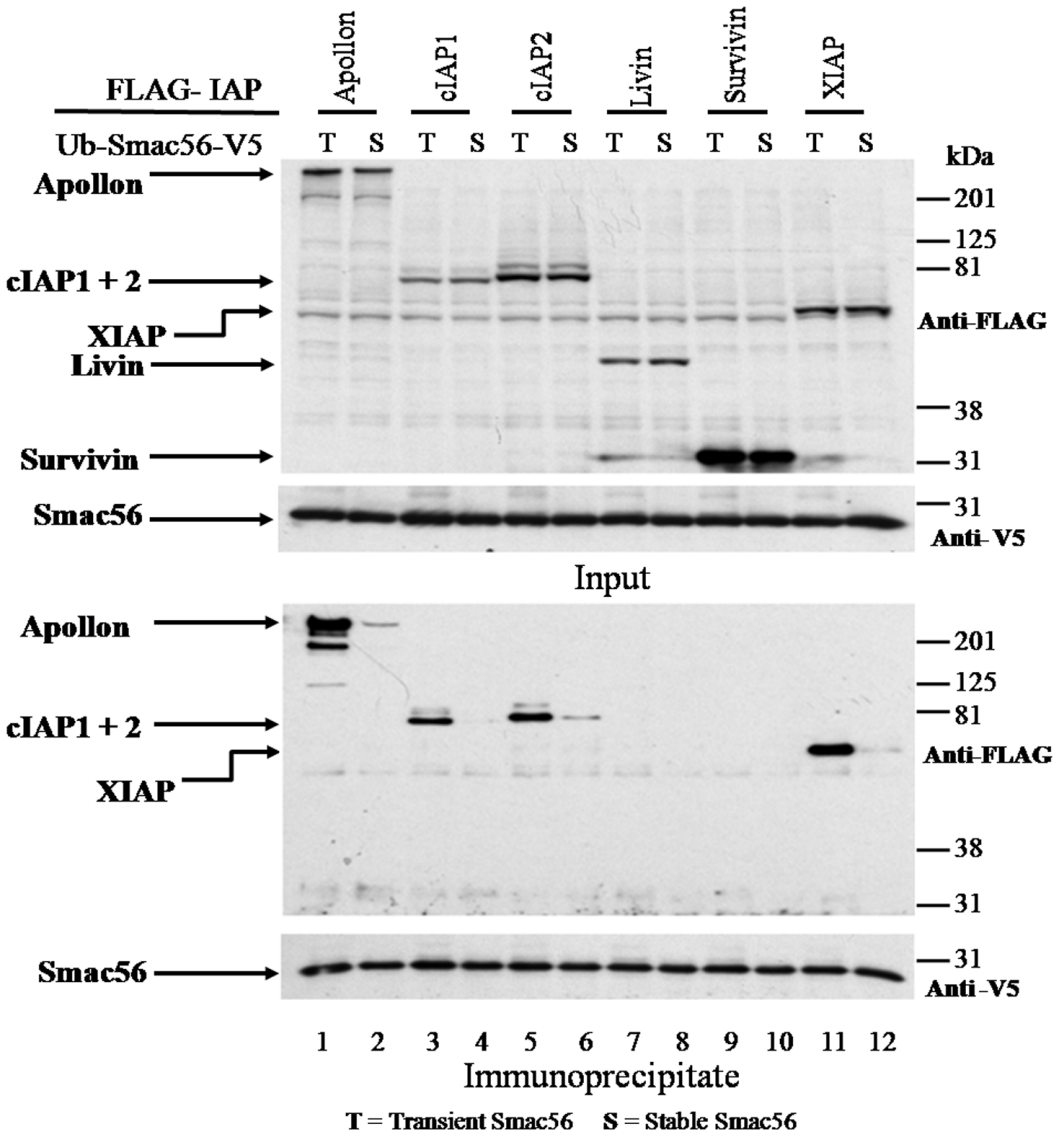
Persistent cytosolic expression of Smac56 attenuates its interaction with Apollon and cIAPs. Cells indicated by a *T* (Transient) were cotransfected with Ub-Smac56-V5 (IRES2.eGFP), whereas those indicated by a *S* (Stable) stably expressed Ub-Smac56-V5 (IRES2.eGFP). Additional cultures were transiently transfected with the indicated FLAG-IAP (p3XFLAG.CMV10). The cells that stably expressed Ub-Smac56-V5 were treated for 12 h with 20 µM zIEALal to increase the level of Smac56, which is rapidly degraded by UPS (Burke and Smith, unpublished data). Cell pellets from each IAP transfection were combined with a pellet of cells that were transiently or stably transfected with Ub-Smac56. The combined cell pellets were lysed, and proteins were immunoprecipitated with a polyclonal antibody to the V5 epitope. Lysate proteins (*Input*) and proteins eluted from the immune complex (*Immunoprecipitate*) were resolved by SDS-PAGE and subjected to western blot analysis with monoclonal antibody to the V5 or FLAG epitope.

Finally, we determined whether stable transfection of cytosolic Smac56-V5 affected the interaction with IAP family members generally. Anti-V5 co-immunoprecipitations were performed on lysates from cells that transiently expressed a FLAG-tagged IAP (Apollon, cIAPs 1 and 2, Livin alpha, Survivin, and XIAP) that were mixed with cells that transiently or stably expressed Ub-Smac56-V5. The cell lines that stably expressed wild type Smac56 were treated with zIEALal for 12 h to increase the level of Smac56-V5 to essentially the same level as that of the transiently transfected cells (Fig. 8, anti-V5, Input). Transiently expressed Smac56-V5 pulled down FLAG-tagged Apollon, cIAP1, cIAP2, or XIAP as indicated by western blot of the co-immunoprecipitates (Fig. 8, lanes 1, 3, 5, and 11, respectively). By comparison to transiently expressed Smac56-V5, stably expressed Smac56-V5 very weakly pulled down FLAG-tagged Apollon, cIAP1, cIAP2, or XIAP (Fig. 8, Lanes 2, 4, 6, and 12 respectively). FLAG-tagged Livin and Survivin failed to pull down either transiently or stably expressed Smac56-V5 (Fig. 8, lanes 7–10). The very weak pulldown of FLAG-XIAP by Smac56 after proteasome inhibition differs from the previously demonstrated undetectable pulldown of stably expressed Smac56-V5 by GST-XIAP from untreated cells (Fig. 5B). We have observed that inhibition of the proteasome evokes accumulation of Smac56 in 911 cells, because UPS efficiently destabilizes Smac56 protein in the cells (Burke and Smith, unpublished data). Possibly some of the nascent Smac56 that is produced during the treatment with the proteasome inhibitor can interact with IAPs and accounts for the minor pull down of Apollon, cIAP1, cIAP2, and XIAP (Fig. 8).

## Discussion

Our results indicate that cytosolic localization of mature Smac can subject it to a novel downregulatory mechanism in 911 line of human embryonic retinoblasts. We have shown that persistent cytosolic expression attenuated the ability of wild type Smac56 to oligomerize and to interact with IAPs (Figs. 5 to [Fig pone-0013094-g006] [Fig pone-0013094-g007] 8). It is known that dimerization defective mutants of Smac fail to interact with BIR2 of XIAP *in vitro* and have a reduced capacity to antagonize XIAP [6]. Interestingly, stably expressed Smac60 retained the ability to dimerize and to interact with IAPs (Figs. 5 to [Fig pone-0013094-g006] 7). The A^60^QKS^63^ residues at the N-terminus of Smac60 may serve as a “secondary” IBM that binds full-length XIAP, as previously suggested [6]. Perhaps the “secondary” IBM accounts in part for the retention of a tenacious interaction between Smac60 and XIAP because the initial Ala of the IBM is critical for IAP binding [6], [19], [40]. It is noteworthy that Smac has a second interaction interface that binds the BIR3 domain of XIAP, which consists of hydrogen bonds and van der Waals interactions and is more than twice the surface area of the IBM-binding interface [7]. Thus, it is not surprising that Smac60 retains the capacity to interact with XIAP *in vitro* [6] and intracellularly (Fig. 1B and 5B). Our Smac60 findings agree well with the study of Smacβ, which showed that the IBM is not required for interaction with XIAP and cIAPs [3], [15].

The Smac dimer is highly stable *in vitro* with a dissociation constant (*K_d_*) of 34×10^−21^ M [11], which may preclude monomer exchange with dimeric Smac. We have found that ectopic Smac56, which was produced in mitochondria from full-length Smac^1-239^, failed to interact with cytosolic Smac56 produced by Ub fusion (Fig. 2). Therefore, cytosolic ectopic Smac56 or Smac60 is unlikely to heterodimerize with endogenous mitochondrial Smac and form a hybrid proapoptotic protein. The extraordinary stability of the Smac dimer supports the idea that the posttranslational modification(s) that attenuates dimerization specifically targets a critical regulatory motif.

Stably expressed cytosolic Smac60, which lacks the IBM and has a weakened ability to antagonize XIAP *in vitro* [6], retained the capacity to complex with XIAP, and to potentiate caspase activation and apoptosis in 911 cells (Figs. 3 to [Fig pone-0013094-g004] 5). Ironically, the potentiation of caspase activation was much greater for Smac60 than for Smac56 (Fig. 3). Furthermore, the proapoptotic function of Smac60 (Figs. 3 and 4) is probably produced by cytosolic homodimeric Smac60, not a heterodimer of Smac60 with endogenous wild type Smac, because we were unable to detect dimerization of cytosolic ectopic Smac56 with mitochondrial Smac56 (Fig. 2). However, the release of endogenous dimeric mitochondrial Smac to the cytosol following proteasome inhibition may contribute to the proapoptotic function of Smac60. The proapoptotic function of Smac60 agrees with the previous report that neither the IBM of Smac, nor the neighboring amino-terminal segment, is required for a proapoptotic function [3]. However, Hunter et al. reported that cytosolic Smac60 failed to potentiate the induction of apoptosis by etoposide in HeLa cells [39]. We have confirmed this finding in 911 cells (Burke and Smith, unpublished data), which indicates that the potentiation of apoptosis by Smac60 depends on the agent used to induce apoptosis.

We have found that the disarming mechanism that monomerized cytosolic Smac56 failed to inactivate Smac60 (Figs. 3 and 5), which indicates that the disarming mechanism depended on the IBM, i.e., the first four amino acids of mature Smac. Presumably a posttranslational modification of one or more residues of the dimer interface near the IBM monomerized Smac. Additionally the disarming mechanism depended on the cellular location of Smac, not on the abundance of ectopic Smac, because the level of cytosolic ectopic Smac was substantially lower than that of endogenous mitochondrial Smac (Fig. 7A). Thus, the disarming mechanism acts on relatively low abundance expression of cytosolic ectopic Smac. Furthermore, the disarming mechanism prefers dimeric to monomeric Smac as discussed below. Studies of a dimer interface mutant support the conclusion that persistent cytosolic expression disables mature Smac dimerization. Stably expressed cytosolic Smac56 comigrated by native PAGE with the ^F88D^Smac56 mutant that is dimerization defective (Fig. 6). By contrast, transiently expressed Smac56 migrated as a doublet of oligomeric bands during native PAGE, similarly to transiently or stably expressed Smac60. Interestingly, we recently observed that endogenous Smac of 911 cells and other human cell lines, which migrates as a single band by SDS-PAGE, migrates as three bands by native PAGE, which correspond in mobility to the monomeric and two oligomeric species of ectopic Smac56 (Burke and Smith, unpublished data). Denaturation of wild type or mutant Smac by 8 M urea or by SDS gave one major Smac species (Figs. 6 and 7). Therefore, the two Smac bands observed by native PAGE differ in mass, presumably oligomeric structure, rather than in charge. Because proteins migrate as a concentrated band by native PAGE, it seems to have greater potential than gel filtration chromatography to detect an additional oligomeric species besides the dimer.

The loss of immunoreactivity of wild type and mutant Smac provided further support for the conclusion that the Smac monomerization involves a posttranslational modification of an amino acid(s) at or near the dimer interface. Thus, certain missense mutations in the dimer interface (V78E, V81D, and F88D) abolished immunostaining by a monoclonal antibody (Fig. 7). Smac monomerization per se did not destroy the epitope because the monomeric L163D mutant was recognized by the antibody (Fig. 7B). Furthermore, the retention of immunoreactivity of the stably expressed L163D mutant suggests that the disarming mechanism prefers dimeric to monomeric Smac. Importantly, transiently expressed wild type Smac56, like transiently or stably expressed Smac60, have an intact epitope (Fig. 7). The segment of the dimer interface that affected epitope recognition (amino acids 78 to 94) has 10 phosphorylatable residues. Addition of a phosphate moiety, like substitution of a hydrophobic residue by an aspartate or glutamate, would be expected to similarly affect recognition by the Smac antibody. Recently Park et al. reported that recombinant Smac can be phosphorylated by JNK3 *in vitro* [41]. Although the stoichiometry of Smac phosphorylation was not reported, phosphorylation decreased Smac interaction with XIAP similarly to our results with Smac56 following persistent cytosolic expression. While we readily detected ubiquitinated Smac56 species following transient cotransfection of Smac56 and XIAP (Fig. 1B), no ubiquitinated Smac56 in cell lines that stably express Smac56 alone was detected (Burke and Smith, unpublished data). We favor the possibility of phosphorylation at or near the dimer interface, which would have little, if any, effect on the mobility of Smac56 by SDS-PAGE as observed (Fig. 5 to [Fig pone-0013094-g006] 7). However, posttranslational modification(s) other than, or in addition to phosphorylation, are not excluded by the present data. An important future achievement will be to identify the posttranslational modification(s) that monomerizes Smac and destroys the epitope recognized by the monoclonal antibody and the enzyme(s) involved.

Curiously, we observed that stably expressed ectopic Smac56, which failed to interact with IAPs (Fig. 8) or to markedly potentiate caspase activation following proteasome inhibition in 911 cells (Fig. 3), potentiated the induction of apoptosis (Fig. 4). Monomeric Smac56 may serve a failsafe proapoptotic function, which is not subject to downregulation via the Ub ligase E3 of IAPs. Furthermore, the proapoptotic function of monomeric Smac56 appears to be independent of IAP antagonism and may not be mediated by caspase activation (Fig. 3C). Because proteasome inhibition slightly increased the level of cytosolic Smac56 that interacted with IAPs (Fig. 8), it is possible that some of the potentiation of apoptosis by Smac56 is due to the canonical IAP antagonism. However, recent studies of dimerization defective Smac mutants indicate that they retain a proapoptotic function that appears to be independent of IAP antagonism (Burke and Smith, unpublished data).

Stably expressed cytosolic Smacδ, which has the IBM but lacks amino acids 62–105, retained the capacity to homodimerize and interact with XIAP as previously reported [4], but failed to heterodimerize with wild type Smac or to potentiate caspase activation or apoptosis (Figs. 2 to [Fig pone-0013094-g003] [Fig pone-0013094-g004] 5). Hence, residues 62–105 of wild type Smac may prevent heterodimerization with Smacδ. Because Smacδ homodimerized (Fig. 5), it was not subject to the same disarming mechanism as wild type Smac. The Smacδ results agree with the Smac antibody data discussed above that indicate the disarming mechanism acts on wild type dimer interface residues (78 to 94) that Smacδ lacks. Stably expressed cytosolic Smacδ failed to potentiate induction of apoptosis evoked by proteasome inhibition, although it modestly increased caspase activation (Figs. 3 and 4). Fu et al. found that transiently expressed mitochondrial Smacδ potentiates apoptosis by chemotherapeutic agents, apparently by decreasing the level of XIAP protein via UPS [4]. Proteasome inhibibition would prevent destabilization of XIAP protein by Smacδ, which may explain the lack of potentiation of apoptosis following proteasome inhibition (Fig. 4). Our results suggest that neither cytosolic Smacβ, which lacks an IBM, nor Smacδ, which homodimerized after stable cytosolic expression (Fig. 5), are subject to the downregulation described here for wild type Smac56. The insensitivity of Smacβ and Smacδ to the monomerizing downregulatory mechanism distinguishes their proapoptotic function from that of wild type Smac.

In summary, the present study uncovered a novel mechanism which can downregulate cytosolic mature Smac. The putative posttranslational modification(s) depended on the presence of the IBM of mature dimeric Smac and occurred at or near the dimer interface.

## Materials and Methods

### Cell culture and transfection

The 911 line of immortalized human embryonic retinoblasts was grown in a 1∶1 mixture of Dulbecco's modified Eagle's medium/Ham's F-12 containing 10% (v/v) fetal bovine serum (FBS) [42]. The cells were plated (6×10^5^ per 35 mm dia. tissue culture dish), incubated for 6 h in a humidified atmosphere containing 5% CO_2_-95% air, and transfected with a modified calcium phosphate method as described [43]. After 16 h, the cells were rinsed with PBS, and refed.

Two days after transfection, the cells were refed with growth medium containing 0.3 mg/ml G418. Two weeks later, green fluorescent protein (GFP) positive cells were sorted and collected with a fluorescent activated cell sorter (FACS). The cells were plated at a low density (100 cells/100 mm dia. culture) to isolate individual colonies. At least three clones were isolated and used in each of the experiments described here. Stably transfected cell lines were always grown in medium containing 0.3 mg/ml G418.

### Western blot analysis

Whole cell lysates were prepared as described for the GST pulldown experiments (see below), and protein concentrations were determined using a Bradford reagent (BioRad). Lysate proteins (20 to 40 µg) were size fractionated by SDS-PAGE. Proteins were electrophoretically transferred to a PVDF membrane and immunostained with antibodies to: the V5 epitope tag (Invitrogen), XIAP (BD Transduction Labs, 610717), GST-π (GS-72, Oxford Biomedical Research, FLAG tag (F9291, Sigma Aldrich), Smac (05-681, Millipore), β-tubulin (T6199, Sigma Aldrich), cleaved PARP (No. 9541, Cell signaling technology), or intact PARP (SA-249, Biomol). Immunostaining was detected with the LumiGLO (Kirkegaard & Perry Laboratories). Exposed X-ray film (B Plus blue, Medlink Imaging) was scanned with an Epson Precision 4870 Photo scanner. Western blots are representative of at least 3 independent experiments.

### Subcellular fractionation

911 cells were plated (4.4×10^6^ per 100 mm dia. culture dish) and transfected with IRES2.eGFP empty vector, or vector encoding Ub-Smac56-V5 or full-length Smac1-239 by a modified calcium phosphate method [43]. Approximately 34 h after transfection, 10 µg/ml cycloheximide was added to the culture media. Six h later the cultures were washed twice with ice-cold PBS, and the cells were detached using a rubber scraper. The cells were collected by centrifugation at 750×g for 5 min at 4°C and resuspended in an equal volume of buffer containing (in mM) 250 sucrose, 20 Hepes-Tris, pH 7.5, 10 KCl, 1.5 MgCl_2_, 1 EDTA, 1 EGTA, 1 dithiothreitol (DTT), and 10 µg/ml each of leupeptin and aprotinin. After a 15 min incubation on ice, the cells were disrupted with a micropipet. Unbroken cells were removed by centrifugation at 750×g for 10 min at 4°C. The mitochondrial pellet was produced by centrifugation at 10,000×g for 15 min at 4°C. The supernatant was centrifuged at 100,000×g for 1 h at 4°C to obtain the cytosolic fraction.

### Pulldown of GST-protein complexes from cell lysates

Cultures were rinsed with ice cold PBS and lysed in a buffer containing 20 mM Tris-HCl, pH 7.4, 150 mM NaCl, 0.2% NP-40, 5 mM DTT, 10 µg/ml leupeptin, 10 µg/ml aprotinin, 0.1 mM sodium orthovanadate, and 80 µM benzamidine. Cells were homogenized by passage through a 26-gauge needle and centrifuged 16,000×g at 4°C for 10 min to remove unbroken cells. The protein concentration of the supernatant was determined with a Bradford reagent (BioRad) using bovine serum albumin (BSA) as a standard. Lysates (0.12 mg protein) were diluted to 1 ml with lysis buffer containing a 25 µl glutathione (GSH)-agarose equilibrated in 20 mM Tris-HCl, pH 7.4, 2 mM MgCl_2_, 1 mM DTT, and protease inhibitors (see above). Lysates were incubated with GSH-agarose for 16 h at 4°C with rotation at 20 rpm. The agarose was washed five times with 1 ml GSH-agarose equilibration buffer. Proteins were eluted with SDS sample loading buffer and resolved by SDS-PAGE.

### Caspase-3-like activity

Cell lines that were stably tansfected with empty vector or Ub-Smac were grown in 35 mm dia. culture dishes, which were inoculated with 5×10^5^ cells. The following day the medium was removed and replaced with culture media with or without 20 µM benzoyloxy-Ile-Glu-Ala-leucinal (zIEALal), and 18 h later attached and detached cell were harvested by scraping in the culture medium. Cell suspensions were centrifuged at 185×g for 5 min. The cells were washed twice with ice cold PBS and collected by centrifugation at 16,000×g for 30 sec. The cells were suspended with a buffer containing 20 mM Tris-HCl, pH 8.0, 1 mM EGTA, 1 mM DTT, and 10 µg/ml leupeptin, aprotinin, and pepstatin. The cells were disrupted by five freeze/thaw cycles using liquid nitrogen and a room termperature water bath. Lysates were clarified by centrifugation at 16,000×g for 10 min at 4°C. The protein concentration of the clarified lysate was determined with a Bradford reagent (BioRad) and BSA as standard. Caspase activity of a 75 µg sample of lysate was assayed with 50 µM acetyl-Asp-Glu-Val-Asp-7-amino-4-methyl coumarin (Ac-DEVD-amc) as substrate. Fluorescence caused by the production of amc was continuously recorded at 440 nm (excitation at 380 nm) at 37°C in 2 ml of buffer containing 20 mM Tris-HCl, pH 8.0, and 2 mM MgCl_2_.

### Flow cytometry analysis of apoptosis and cell death

Stably transfected cells were plated at 1.2×10^6^ cells per 60 mm dia. culture dish. The following day the cultures were refed with fresh growth medium with or without 20 µM zIEALal, a peptidyl proteasome inhibitor. Twenty-four hours later the floating and attached cells were scraped into the growth medium and harvested by centrifugation at 185×g for 5 min at room temperature. The cells were washed once with ice cold PBS, suspended with 0.05% trypsin in PBS, and incubated for 5 min at room temperature. The trypsin reaction was stopped by addition of 1 ml growth medium containing 10% FBS. Cells were centrifuged at 2,300×g for 30 s at room temperature and washed twice with Annexin V-PE binding buffer: 10 mM Hepes/NaOH, pH 7.4, 140 mM NaCl, 2.5 mM CaCl_2_. The cells were suspended in 1 ml of the binding buffer and 5 µl Annexin V-PE (BD Pharmingen) and 5 µl 7-amino-actinomycin D (7-AAD) viability staining solution (eBioscience) were added. The cells were incubated at room temperature in the dark for 15 min, collected by centrifugation, and suspended with 1 ml binding buffer. The cell suspensions were filtered using cell-strainer cap tubes (Falcon 352235) and analyzed within 1 h of beginning the incubation with Annexin V-PE using a BD LSR II FACS.

### Native and urea PAGE

For native electrophoresis, polyacrylamide gels (10%) were prepared as described by Sambrook et.al [44] except SDS was omitted from the gel, the sample loading buffer, and the running buffer. Similarly, 10% gels (1 mm thick) containing 8 M urea were prepared and proteins electrophoresed without SDS using the mini-PROTEAN 3 system (Bio-Rad). Lysate proteins (5 to 20 µg) from cells stably or transiently transfected with wild type or a Smac variant were resolved on the gels to normalize ectopic protein levels. Lysates were loaded onto native gels with an 8 times concentrated loading buffer: 400 mM Tris HCl, pH 6.8, 5.7 M β-mercaptoethanol, 80% glycerol, and 0.05% bromophenol blue. Lysates for urea gels were incubated at 4°C for 1 h with 2 times concentrated loading buffer; 8 M urea, 50 mM Tris-HCl, pH 6.8, 715 mM β-mercaptoethanol, and 0.006% bromophenol blue, prior to electrophoresis. Samples for both gel types were loaded without boiling, to reduce the possibility of protein aggregation. Gels were run at a constant 175 V for 90 min at 4°C in a buffer containing 25 mM Tris and 192 mM glycine buffer. Proteins were transferred to a PVDF membrane with the usual SDS transfer buffer, and western analysis was performed.

### Co-immunoprecipitation of IAPs with Smac56-V5

911 cells (6×10^5^ cells per 35 mm dia. dish) were transiently transfected with IRES2.eGFP Ub-Smac56 or p3XFLAG.IAP plasmids. The following day transiently transfected cultures were refed with fresh growth media, and cultures of stably transfected IRES2.eGFP Ub-Smac56-V5 cells were plated (8×10^5^ cells per 35 mm dia. dish) and allowed to incubate for 12 h before refeeding with medium containing 20 µM zIEALal. The stably and transiently transfected cultures were washed twice with ice-cold PBS, and the cells were scraped from the dishes in PBS. Cell suspensions containing each of the transiently transfected FLAG-IAP cells were combined with cells that transiently or stably expressed Smac56. The combined cell suspensions were centrifuged at 16,000×g for 15 s at 4°C, and the cells were lysed in immunoprecipitation buffer containing 150 mM NaCl, 20 mM Tris-HCl, pH 7.5, 0.2% NP-40, 3 mM MgCl_2_, 1 mM EDTA, 1 mM EGTA, 10 µg/ml each of leupeptin and aprotinin, 0.1 mM sodium orthovanadate, 80 µM benzamidine, and 100 µM pefabloc SC. A sample of each lysate (30 µg) was subjected to western blot analysis, and the remainder of each lysate was diluted to 1 ml with lysis buffer, and 0.3 µg of a polyclonal V5 antibody (AB3792, Millipore) was added. After 2 h at 4°C, 10 µl of buffer equilibrated protein A/G plus agarose (Sc-2003, Santa Cruz) was added and the incubation continued for 1 h. The protein A/G agarose was washed 3 times with 1 ml lysis buffer, and immunoprecipitated proteins were eluted with SDS sample loading and subjected to western blot analysis with monoclonal antibodies to the FLAG and V5 epitopes.

### Ub-Smac fusion constructs

Total RNA was prepared from HeLa cells using Trizol reagent (Gibco BRL) according to the instructions of the manufacturer. Total RNA (1 µg) was incubated with 10 pmole Smac reverse primer (see Table 1 for all primer sequences) in a total volume of 14 µl at 70°C for 5 min. The reaction was immediately placed on ice and the following were added: 1 µl RNAse Inhibitor (Eppendorf), 5 µl MMLV RT buffer, 1 µl MMLV RT (200 units/µl) (Promega), and 4 µl 10 mM dNTPs. The reaction was incubated at 42°C for 1 h, followed by 15 min at 70°C. A sample (2 µl) of the reaction was used to amplify full-length Smac using the full-length Smac forward primer and the Smac reverse primer with the following PCR cycling conditions: 94°C for 1 min; followed by 30 cycles of 94°C for 30 s, 65°C for 30 s, and 72°C for 2 min; and finally 72°C for 5 min. The PCR product was cloned into pCRBLUNT (Invitrogen).

**Table 1:** Primers used for cloning and overlap extension.

Smac cloning and Overlap extension primers	
Primer name	Primer sequence
Smac reverse	TTTGCGGCCGCCATCCTCACGCAGGTAGG CCTCCTGCTC
Full-length Smac forward	CCCAAGCTTGCAATGGCG GCTCTGAAGAGTTGGGCTGTCG
HA-Ub forward	GGGAAGCTTGCAAT GGCTAGCTACCCTTATGACGTCCCCG
Ub-Smac overlap forward	GTCTCAGAGGTGGGATGGCGGCTCTGAAGAGTTGGCTGTC
Ub-Smac overlap reverse	TTCAGAGCCGCCATCCCACCTCTGAGACGGAGGACCAGGT
Ub-56-239 forward	CGTCTCAGAGGTGGGGCGGTTCCTATTGCACAGAAATCAGAG
Ub-56-239 reverse	TGCAATAGGAACCGCCCCACCTCTGAGAC GGAGGACCAGGT
Ub-60-239 forward	CGTCTCAGAGGTGGGGCACAGAAATCAG AGCCTCATTCCCTT
Ub-60-239 reverse	CTCTGATTTCTGTGCCCACCTCTGAGACGG AGACGGAGG ACCAGGT
Smacδ forward	CGGTTCCTATTGCACAGGCTGTTTATACCTTAACTTCTCTTTACCG
Smacδ reverse	TAAGGTATAAACAGCCTGTG CAATAGGAACCGCCCCACCTCT
Ub forward BamHI	GGCGAATAGGATCCAGAGGCATGCAGATCTTCGTGAAG
V5 reverse Bam HI	GGCTTAGTGGATCCTTAACGCGTAGAATCGAGACCGA
Ub forward ClaI	GAATTCATCGATGCAGATCTTCGTGAAGACCCTGACTGG
Smac reverse ClaI	GAATTCATCGATGAATCCTCACGCAGGTAGGCCTC
**pGFLEX XIAP**	
XIAP forward	GCGAGCGGCCGCATATGACTTTTAACAGTTTTGAAGGAT
XIAP reverse	GGGGAGCGGCCGCTAATTAAGACATAAAAATTTTTGC
**Mutagenesis primers**	
Smac V78E forward	GTGAAGCATTGATGAGGAGAGC AGAGTCTTTGGTAACAG
Smac V78E reverse	GAGGTGCTATCTGTTACCAAAGACTCTGCTCTCCTCATC
Smac V81D forward	GATGAGGAGAGCAGTGTCTTTGGACACAGATAGCACCTC
Smac V81D reverse	GAGAAAGGTAGAGGTGCTATCTGTGTCCAAAGACACTGC
Smac F88D forward	ACAGATAGCACCTCTACCGACCTCTCTCAGACCACATATGCG
Smac F88D reverse	CGCATATGTGGTCTGAGAGAGGTCGGTAGAGGTGCTATCTGT
Smac L163D forward	CTTGGATGACTGCAGTTGGTGACTCAGAGATGGCAGCAGAAG
Smac L163D reverse	GCAGCTTCTGCTGCCATCTCTGAGTCACCAACTGCAGTCATCC
Ub G76V forward	GTCCTTCGTCTCAGAGGTGTGGCGGTTCCTATTCACAG
Ub G76V reverse	CTGTGCAATAGGAACCGCCACACCTCTGAGACGAAGGAC
**3xFLAG IAP primers**	
cIAP1 forward	CACCATGCACAAACCTGCCTCCCAAAGAC
cIAP1 reverse	TCATTAAGAGAGAAATGTACGAACAG
cIAP2 forward	CACCATTGAACATAGTAGAAAACAGC
cIAP2 reverse	TCATCATGAAAGAAATGTACGAACTGTACC
Livin forward	CACCATGGGACCTAAAGACAGTGCC
Livin reverse	TCACTAGGACAGGAAGGTGCGCACGCG
Survivin forward	CACCATGGGTGCCCCGACGTTGCCC
Survivin reverse	TCATCAATCCATGGCAGCCAGCTGCTC
XIAP forward	CACCATGACTTTTAAGAGTTTTGAAG
XIAP reverse	TTATTAAGACATAAAAATTTTTTGCTTG

A pcDNA3.1 plasmid (Invitrogen) encoding Ub with an amino-terminal hemagglutinin (HA) tag was used as a template to amplify HA-Ub [43]. The full-length Smac1-239 cDNA was excised from pCRBLUNT using Hind III and Not I, and subcloned into pcDNA3.1/V5-His B. HA-Ub-Smac cDNA was generated by two-step overlap extension. The first step consisted of two reactions to amplify HA-Ub and full-length Smac separately using the following cycle conditions: 94°C for 1 min; 30 cycles of 94°C for 30 s, 65°C for 30 s, and 72°C for 2 min; and 72°C for 5 min. In the second step, a sample (2 µl) of the PCR products from each reaction was mixed and reamplified: 94°C 1 min; 25 cycles of 94°C for 30 s, 65°C for 30 s, 72°C for 2 min; and 72°C for 5 min. The final products were digested with Hind III and Not I, purified with QIAquick PCR purification kit (Qiagen), and ligated into pcDNA3.1/V5-HisB. The HA-Ub forward and the Smac reverse primers were used for all second step reactions. The following overlap extension primers were used: Ub-Smac overlap forward primer, and Ub-Smac overlap reverse primer. A plasmid encoding HA-Ub-Smac1-239 was used as a template to generate the HA-Ub-Smac56-239 and HA-Ub-Smac60 fusions by overlap extension PCR using Ub-Smac56-239 forward and reverse primers and HA-Ub-Smac60 forward and reverse primers, respectively. The HA-Ub-Smac56-239 plasmid was used as a template to generate HA-Ub-Smacδ by overlap extension PCR using HA-Ub-Smacδ forward and reverse primers. Smac56 and G76VUb-Smac56 point mutants were generated by the QuikChange method (Stratagene) with the protocol suggested by the manufacturer. The following pairs of forward and reverse primers were used to produce the indicated amino acid substitutions: V78E; V81D; F88D; L163D; and UbG76V. The pcDNA3.1 expression plasmids were used as templates to PCR amplify and subclone the Ub-Smac inserts lacking the HA and His tags into IRES2.eGFP (Clontech) after BamHI digestion. The following primers were used: Ub forward BamHI and V5 reverse BamHI. Ub-Smac56 and Ub-Smacδ were cloned in frame with a C-terminal glutathione sulfotransferase (GST) tag in pGFLEX (ATCC 87629) after PCR amplification with the following primers: Ub-forward ClaI, and Smac reverse ClaI. The PCR product and the pGFLEX plasmid were digested with ClaI. The digested plasmid was dephosphorylated and ligated with the PCR product. Clones with the insert in the forward orientation were identified by nucleotide sequence analysis. All cDNA inserts were validated by complete nucleotide sequence analysis.

### Gateway cloning of IAPs into destination vectors

A CMV10 expression plasmid with a 3xFLAG-tagged Apollon insert was generously provided by M. Naito [29]. Plasmids containing cDNA inserts of cIAP1 (MGC-26517), cIAP2 (10436078), Survivin (MGC-8592), and Livin (10659534) were purchased from ATCC. A pGFLEX-XIAP plasmid was produced as described below. The plasmids were used as templates to amplify and subclone the IAPs into the pENTR/D-TOPO plasmid (Invitrogen). The following forward and reverse primer pairs were used in the PCR reactions: cIAP1; cIAP2; Livin alpha; Survivin; and XIAP. Plasmids containing the appropriate insert were identified after restriction endonuclease digestion and confirmation of the nucleotide sequence. A gateway compatible p3xFLAG-CMV10 (Sigma) destination plasmid was created after Eco RV digestion and in frame ligation of a recombination site, using the Gateway vector conversion system (Invitrogen) according the manufacture's protocol. The pENTR/D-TOPO (Invitrogen) plasmids containing IAPs were mixed with a p3xFLAG destination plasmid or the pDEST-15 plasmid (Invitrogen) for bacterial production of GST-IAPs. Recombinant plasmids were produced by incubation in a reaction containing LR Clonase II. Proper recombination was confirmed by PCR amplification with gene specific primers for each IAP. All cDNA inserts were validated by complete nucleotide sequence analysis.

### Cloning of XIAP into GST expression plasmids

pcDNA3.1/GS XIAP (Invitrogen) was used a template to PCR amplify XIAP with forward and reverse XIAP primers primer with the following PCR cycling conditions: 94°C for 1 min; followed by 30 cycles of 94°C for 30 s, 65°C for 30 s, and 72°C for 2 min; and finally 72°C for 5 min. The product was ligated into pCRBLUNT, excised with Not I and subcloned into pGFLEX. The pGFLEX.XIAP expression plasmid was used for mammalian expression of GST-XIAP.
